# Ampullaviruses: From Extreme Environments to Biotechnological Innovation

**DOI:** 10.2174/0113892010325244240916112436

**Published:** 2024-09-27

**Authors:** Alaa A. A. Aljabali, Mohamed El-Tanani, Almuthanna Alkaraki, Vijay Mishra, Yachana Mishra, Murtaza M. Tambuwala

**Affiliations:** 1 Faculty of Pharmacy, Department of Pharmaceutics & Pharmaceutical Technology, Yarmouk University, Irbid 21163, Jordan;; 2 College of Pharmacy, Ras Al Khaimah Medical and Health Sciences University, Ras Al Khaimah, UAE;; 3 Department of Biological Sciences, Faculty of Science, Yarmouk University, Irbid 21163, Jordan;; 4 School of Pharmaceutical Sciences, Lovely Professional University, Phagwara (Punjab)-144411, India;; 5 School of Bioengineering and Biosciences, Lovely Professional University, Phagwara (Punjab)-144411, India

**Keywords:** Ampullaviruses, virophage, archaeal viruses, host-virus interactions, pharmaceutical biotechnology, viral nanotechnology

## Abstract

Ampullaviruses are unique among viruses. They live in extreme environments and have special bottle-shaped architecture. These features make them useful tools for biotechnology. These viruses have compact genomes. They encode a range of enzymes and proteins. Their natural environment highlights their suitability for industrial applications. Ongoing research explores ways in which these viruses can improve enzyme stability. They are also employed in the creation of new biosensors and the development of new bioremediation techniques. High co-infection rates and the ecology of ampullaviruses at larger scales can also reveal new viral vectors. They can also help improve phage therapy. Here, we have explored the structure and function of ampullaviruses. We have focused on their use in biotechnology. We have also identified their characteristics that could prove to be useful. We have also pointed out key knowledge gaps and bridging them could further extend the biotechnological uses.

## 1 INTRODUCTION

The last decade has been marked by outright revolutions in biotechnology. Consequently, scientists are constantly on the lookout for new tools and techniques that could help to improve various fields of study [1]. Among these promising tools, ampullaviruses have emerged as potential vectors for gene editing, biocontrol, and diverse applications in biotechnology. Ampullaviruses are giant double-stranded DNA viruses characterized by their peculiar bottle-shaped morphology. In addition, they possess complex mechanisms of replication, which set them apart from other groups of viruses [2,3]. These unique characteristics have sparked significant interest in the scientific community, prompting extensive research into their potential biotechnological applications. The very same attributes make the ampullaviruses interesting objects for basic virology research and hold this virus group as great candidates for a whole range of biotechnological applications where large genomes, giving ample space for genetic engineering, exhibit peculiar mechanisms of replication that promise advantages in gene delivery and expression systems.

Of particular interest are the enzymes encoded by ampullaviruses. These viruses host heat-stable enzymes from extremely hot environments. Such enzymes could be useful in high-temperature industrial processes, including specific fermentation and biofuel production. Although researchers have identified these enzymes, they have not yet been isolated or described for practical use.

Ampullaviruses infect archaeal hosts through unique mechanisms. These mechanisms can help create new expression systems for extremophilic archaea. These systems could help express proteins that are hard to produce in traditional hosts. This application shows promise. However, it requires much more information about how ampullaviruses interact with their hosts. But still, this could only be speculation. Currently, no one has attempted to create nanoparticles by employing ampullaviruses. No biotech process has used them until now. The extreme conditions associated with ampullaviruses make it hard to use these materials in a standard lab or industry. Despite these challenges, ongoing research on ampullaviruses improves our understanding of viral diversity. It also enhances our knowledge of extremophile biology and limits. While this knowledge has limited immediate applications, it may prove to be valuable.

This review puts an emphasis on updating the structure, the life cycle, and possible biotechnological applications of ampullaviruses to provide a more structured overview. A better understanding of these features may reveal their contributions to genetic engineering and medicine. The review aimed to provide an overview of ampullaviruses. It emphasizes their biotech applications. We have summarized recent findings on their structure and genome. We have also discussed as to how they interact with hosts. We have assessed their usefulness in editing genes and delivering medicines. We have brought together structural biology, genomics, and biotechnology insights. We have also offered a new view of oddball viruses' potential. They could prove to be significant tools in areas from basic research to applied biotechnology.

While in general, viruses have been used successfully to a great degree in different biotechnological applications and are, therefore, very versatile with huge potential across different fields, geminiviruses, as described, are efficient actors in the manipulation of cell functions and provide several strategies that can be exploited for biotechnological means. Plant viruses and their derived virus-like particles have already been put to many uses in biotechnology, and have proven to be important tools in this field. Additionally, the genome structure of viruses has been recognized to be complete with appropriate machinery for use as an efficient and useful tool in biotechnology applications within medicine, the material industry, and agriculture as a whole [4,5]. Further, the cowpea mosaic virus has found applications in biotechnological and nanotechnological uses. This turns out to be the proof for the applications of viruses in different fields for a long time [6,7].

The thermophilic viruses represent a new source of genetic material and enzymes that hold enormous potential for use in biotechnology, which means that there are many resources to be exploited for biotechnological advancement [8]. Virus-like Particles (VLP)- and Virus Nanoparticles (VNP)-based plant nanotechnologies are some of the most promising platforms for a variety of biotechnological applications in medicine and beyond. In this respect, many Cowpea Mosaic Virus (CPMV)-based biotechnological and nanotechnological applications have already been reported, highlighting the long history of viruses in these fields of application [9-12].

Recent trends have significantly advanced biotechnology as researchers have explored novel tools and techniques. Ampullaviruses have attracted considerable attention due to their potential applications. Giant double-stranded DNA viruses have shown promise in gene editing and biocontrol, making them important for biotechnological research [13]. This review has focused on ampullaviruses and biotechnological research. Their structure, life cycle, and potential applications have been examined to provide insights into their contributions. These characteristics have been found to make ampullaviruses unique among viruses and valuable for genetic engineering. The giant bottled-shaped morphology and complex replication mechanisms, which differ from those of all other known groups, have paved the way for new biotechnological approaches [14]. Molecular details of ampullaviruses may lead to breakthroughs in gene delivery, vaccine design, and gene therapy. Understanding the interactions between ampullaviruses and their hosts can be crucial to determining their benefits in diverse biotechnological fields, such as genetic engineering and medicine.

Ampullaviruses belong to the family of viruses known as *Ampullaviridae*, consisting of viruses with an icosahedral bottle-shaped capsid and a linear double-stranded DNA genome with terminal inverted repeats [15]. They infect hyperthermophilic archaea of the genus *Acidianus*, with genome size spread ranging from 5–50 kb. The host's replisome is involved in the ABV-to-host takeover, which is facilitated by specific genes. The only known cultivated representative, *Acidianus* Bottle-shaped Virus (ABV), has been found in a hot acidic spring in Pozzuoli (Italy). It showed a limited host range and did not induce host cell lysis [16-18]. This viral family has been found to have unique and interesting properties [16]. Ampullaviruses specifically infect hyperthermophilic archaea, particularly those belonging to the genus *Acidianus*. The viral takeover of the host replisome is aided by a specific set of genes that facilitate viral uptake.

ABV's genome includes 57 proteins, DNA polymerases, and other functional units. Phylogenetic relationships show the best affiliation between ABV2 and ABV, with weak sequence similarity to ABV3. This suggests that ampullaviruses may be among the most ancient forms of hyperthermophilic archaeal viruses, with a distinct evolutionary history [16, 17]. Ampullavirus research covers structural to functional, environmental, and evolutionary genomics, including a critical association between transposons and gene exchange in *Euryarchaeal* plasmids. Recently, interdisciplinary studies have used genomic tools for phylogenomic analyses and the characterization of host-associated LAB strains for putative ampullaviruses and their hosts. Ampullaviruses have been found to be promising for biotechnological and probiotic applications [19,20]. Additional research into genomics, biotechnological applications, and related areas is critical for an in-depth study of these viruses. However, our understanding of ampullavirus evolution is limited by the lack of cultivated specimens. The virions of ampullaviruses have a unique shape, measuring 230 ± 20 nm, and tapering from a broad end width of 75 nm to a narrow end of 4 ± 1 nm [5].

The characteristic appearance of the virus is attributed to the fact that the broad end of the virion is studded with a ring of 20 slender filaments, each measuring 20×3 nm and equally spaced [16]. In addition to its distinctive appearance, a ring of 20 evenly spaced filamentous structures measuring 20 × 3 nm decorates the larger ends of the virions [21]. The virions are enveloped with a fine 9 nm thick envelope, and within this is a funnel-shaped protein coat, providing protective housing for the viral DNA [22]. The genome of an ampullavirus is a linear double-stranded DNA of approximately 23.8 kb, which distinguishes it from viruses that infect bacteria and eukaryotes [23]. Another one in the line of features drawing attention towards ampullaviruses is the feature of bottle-shaped morphology, and there has been quite a lot of speculation about possibly the most critical aspect in the introduction of viral DNA into the host cell *via* the virion's narrow end.

The genome of an ampullavirus is a linear double-stranded DNA of approximately 23.8 kb, a feature that distinguishes it from viruses that infect bacteria and eukaryotes [23]. Another notable feature of ampullaviruses is the feature of bottle-shaped morphology. There has been significant speculation about the virion's narrow end, possibly the most critical aspect in the introduction of viral DNA into the host cell. This unique genomic organization characterizes ampullaviruses, leading to fascinating speculations about the nature of the virus-host relationship and molecular mechanisms involved in infection [24,26]. A unique structural arrangement sets ampullaviruses apart, which are beyond those of viruses with target-specific targets, either bacterial or eukaryotic hosts [25,26].

Ampullaviruses are characterized by their ampulla-shaped capsids and large genomes, and infect a wide range of hosts across bacterial, archaeal, and eukaryotic domains. Recent genomic studies have revealed significant biological adaptations in ampullaviruses, including a large number of functionally uncharacterized genes, suggesting that the biological potential of this clade remains largely unexplored [20]. Promising biotechnological applications for ampullaviruses include capsids as potential candidates for novel drug delivery systems and the use of genomes as possible sources for novel gene therapy vectors and industrial enzymes [26,27]. Current knowledge primarily associates ampullaviruses with *Bacteroidetes*. Ongoing research aims to discover their potential in biotechnology, particularly in phage therapy and biocontrol.

The literature search has revealed diverse biotechnological potentials for different microorganisms, deviating from the focus on ampullaviruses [28,29]. Genomic analyses of *Weissella*, *Nesterenkonia*, and *Microvirga* have highlighted their applications in fermentative processes, cold adaptation, plant growth promotion, and the production of pharmaceutical and industrial secondary metabolites. Discussions on antimicrobial peptide production in plants and the use of *Ustilago maydis* as a yeast model have further underscored the wide range of biotechnological prospects across microorganisms [30-32]. The biotechnological potential of ampullaviruses is intriguing given their unique capsids and constantly evolving genomes. However, the differences in search results have indicated the need for more targeted research on ampullaviruses. An interdisciplinary approach is necessary to fully understand their evolutionary context and potential applications. Thus, more detailed studies should be conducted specifically on ampullaviruses to reveal the full biotechnological potential of this group [33,34].

This review has examined the latest genomic studies on ampullaviruses, their unique bottle-like morphology, and their biotechnological potential among diverse viral families. Ampullaviruses exhibit a distinct form, notably bottle-shaped, rather than the typical helical or icosahedral shape of other viruses. This review has discussed the features of this unique structure in ampullaviruses and their possible roles in biotechnology. The recently discovered family of ampullaviruses is defined by unusual bottle-shaped virions that thrive in extreme environments.

The taxonomic classification of ampullaviruses can be divided into three categories: family, genus, and species. Further studies are needed to understand the biotechnological potential and peculiar structure of ampullaviruses, which can help to explore new research opportunities and application prospects. However, to fully exploit these benefits, their genomes and mechanisms must be better clarified. Additional research is necessary to explore and identify the full potential of this diverse group of viruses [10].

The family *Ampullaviridae* is the top taxon in the taxonomy of viruses, and includes all known ampullaviruses. Members of this taxonomic family possess a few unique characteristics that define them as ampullaviruses, such as the bottle-shaped architecture of their virions [16]. This adaptation points to the adaptability and robustness of these viral entities. At the species level, ampullaviruses are rigorously classified according to their genomic features and the host archaea they infect. This classification ensures that all identified species belong to unique clusters of viruses with common genetic traits, allowing for proper scientific understanding and classification within the genus *Ampullavirus* [18,25].

Specific genomic signatures characterize each of these species and are better adapted to one of the extreme habitats, contrasting with another, which again points to the adaptability and robustness of the viral entities [34]. Despite their wide distribution and vital status, ampullaviruses are difficult to study and exploit, and thus, they remain mysterious. Recent technological advances have created opportunities for in-depth studies, and have made ampullaviruses a new hotspot for virology research. These viruses have been among the oldest groups of viruses, which have co-evolved with bacteria for millions of years, and thus, have had a significant influence on bacterial genome evolution [35,36].

Ampullaviruses have an atypical bottle-like shape, with a tail structure resembling a flask or ampulla. This unique morphotype has recently attracted worldwide scientific interest due to potential applications in viral assembly, biotechnological applications, and structural biology [37]. Beyond these morphological characteristics of ampullaviruses, the knowledge of the life cycle of ampullaviruses is important for more comprehensive insight into archaeal viruses and the general relationship between viruses and hosts. The combined approach involving morphology and life cycle can enable the formation of detailed descriptions, going beyond generally the typically described helical or icosahedral structures [19].

The unique structural characteristics of ampullaviruses described here, along with their genomic data, provide the basis for understanding their biology and exploring their potential applications in other scientific fields. This review has given special consideration to the distinctive structural features of ampullaviruses, which are neither helical nor icosahedral. It has also examined the biotechnological applications of ampullaviruses, including their advantages and drawbacks [23,34].

This review has further explored the structural features of ampullaviruses, differing from the classical helical or icosahedral forms of most other viruses [38]. These unique morphologies have become the subject of extensive research. The review has also described the biotechnological landscape of ampullaviruses based on foundational references and current research. It has examined how ampullaviruses can be used for biotechnological applications, including their potential benefits and challenges.

**Fig. (1):**
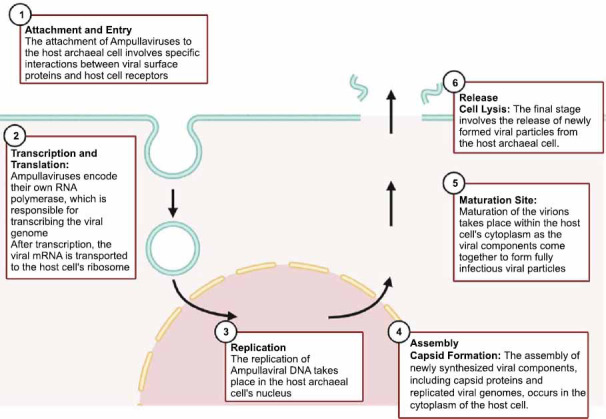
Ampullaviruses: A schematic representation of the ampullavirus life cycle, including the attachment, entry, replication, assembly, and release stages. In the face of such a fine-scaled life cycle, knowledge of this cycle is indispensable for understanding the dynamics of archaeal virus-host interactions, and for potential applications in biotechnology and beyond.

Further research into the biotechnological potential of ampullaviruses can be conducted by taking into account genomics and structural biology, as well as the life cycle, which can open up applications in the fields of biotechnology and medicine. The molecular mechanisms underlying the interaction between ampullaviruses and their hosts have to be understood in order to unlock these unique properties for genetic engineering and innovative therapeutic solutions [27,37]. We have, herein, also looked at research in progress on genomic, structural, and life cycle features of ampullaviruses, necessary to reach their full biotechnological potential (Fig. **1**).

## 2 UNRAVELING THE UNCONVENTIONAL MORPHOLOGY

Ampullaviruses have unique potential for versatile biotechnological applications, especially in targeted drug delivery, gene therapy, and vaccine developments [39-41]. Ampullaviral capsids exhibit extraordinary stability; thus, they have become an advantageous approach for designing drug delivery systems [42]. Recent studies on genomics have revealed quite a number of exciting areas in which ampullaviruses could be applied. This is especially in regard to their unique capsids, which make them potential novel drug delivery systems and gene therapy vectors, and their genomes, which could express some industrial enzymes. In even greater depth, the interactions between ampullaviruses and their hosts could give way to breakthroughs in gene delivery or the design of vaccines [43,44].

The advantages of using ampullaviruses in targeted drug delivery include the following:

1. Nanoscale engineering: Encapsulation efficiency of nanoscale scaffolds can lead to engineered viruses for various cargoes, specifically nucleic acids, proteins, peptides, and drugs [45,46].
2. Stability: The high stability of viruses under different body conditions makes them ideal for drug delivery and bioimaging applications [47,48].
3. Viruses can be easily produced on an industrial scale, which is a critical requirement for their effective use in drug delivery and other biotechnological processes [12,49]. Viruses can be easily produced on an industrial scale, which is a critical requirement for their effective use in drug delivery and other biotechnological processes [12,49].
4. Targeting ligands or peptides: It easily manipulates genetically or chemically the outer surface of Viral-like Particles (VLPs) originating from ampullaviruses, which can easily accommodate targeting ligands or peptides [50,51].
5. Drug encapsulation: Many VLPs, particularly ampullaviruses, encapsulate hydrophobic drugs to facilitate their exact delivery at specific sites [52,53].

### 2.1 Targeted Drug Delivery Potential

Ampullaviruses enter the host cell by membrane fusion, bypassing endosomal pathways. This could theoretically enhance the delivery of the therapeutic cargo [54]. Limited information is available regarding the host range specificity of ampullaviruses. However, some evidence suggests that ampullaviruses likely infect specific bacterial hosts. This characteristic may be useful in developing targeted gene delivery systems.

The capsid structure of ampullaviruses is suitable for decoration with targeting ligands, enabling specific delivery of therapeutic genes to particular cell types or tissues. The dsDNA genome of ampullaviruses allows for various types of genetic manipulation, including the insertion of therapeutic genes and deletion of non-essential or deleterious viral genes [55]. Ongoing studies are investigating these systems.

While ampullaviruses show promise, other viral-like particles, such as adenoviruses, lentiviruses, and retroviruses, have developed genetic mechanisms to ensure the efficient transduction of target cells [56-58]. Drug delivery systems using Virus-like Particles (VLPs) and nanostructures, such as those from Human Papillomavirus (HPV) and bacteriophages, can be functionalized with ligands targeting human cancer cell surface receptors to achieve targeted drug delivery to cancer cells [45,59].

While the exact potential of ampullaviruses in drug delivery requires further investigation, their bottled shape makes them promising candidates. The unique morphology of ampullaviruses presents novel candidates for drug-carrier development. Its narrow end is expected to mediate the delivery of viral DNA; hence, it also offers the potential for the delivery of targeted therapeutic agents. The narrow end of the virus is thought to mediate the delivery of viral DNA, suggesting potential for the delivery of targeted therapeutic agents. These unusual structural characteristics have not been thoroughly explored for drug delivery. However, ampullaviruses should be considered for development as potential targeted delivery systems [60,61].

Significant safety concerns include immune responses, genotoxicity, and off-target effects. Knowledge gained from other viral vectors can be applied for the safe development of ampullavirus-based gene delivery systems [62]. It is crucial to develop an effective process for ampullavirus production and purification for use in gene therapy. Cell culture systems and purification methods used for other viral vectors, both small and large, can likely provide valuable information that can be applied when designing production processes to reach the required manufacturing scale [62]. Although research on ampullaviruses is still in the early stages, they are considered promising candidates as gene delivery vectors for many applications, primarily due to their features [63]. Therefore, understanding the mechanisms of entry, host interactions, and manipulation options is required to exploit these benefits, which can significantly improve gene therapy approaches, leading to more targeted and efficient therapy for patients [63].

### 2.2 Gene Therapy Potential

Research suggests a possible application of ampullavirus genomes in gene therapy. Ampullavirus genomes are large, allowing them to carry several genes. This makes them potentially useful for developing gene therapy technology [64]. Key features that make them valuable for gene therapy include their large genomic size, which can hold many genes at once, and their ability to code for multiple proteins, which can enable the transfer of several therapeutic elements simultaneously. While few direct applications for gene therapy are currently known, the basic genomes of ampullaviruses offer some advantages [65]. The limited number of reported cases highlights the need for more research to gain a complete picture of ampullavirus genomes. This understanding could help develop new gene therapy vectors.

### 2.3 Vaccine Development

Ampullaviruses might be useful for developing vaccines based on their unique capsids. Important factors for using ampullaviruses in vaccination include the following: the ampullavirus capsid needs to be stabilized to maintain the integrity of the viral particles during delivery. Also, ampullaviruses must efficiently deliver the antigen to achieve better immunogenicity [66]. No specific cases are available with respect to the use of ampullaviruses in vaccine development; however, the intrinsic properties of ampullavirus capsids and their potential to produce a potent immune response suggest their viability in this biotechnological application [67].

Ampullaviruses may be a better choice of vectors for vaccine production, despite few studies on them. Conversely, the non-integrating nature of this type of dsDNA virus could reduce the risk of insertional mutagenesis, potentially making it safer for vaccine production than integrating viral vectors. These viruses also have unique protein capsids and potential membrane structures that are likely to induce a robust immune response in both humoral and cellular environments, which is critical for vaccine efficacy [68]. Additionally, ampullaviruses could present an effective platform for targeted delivery to specific immune cells, particularly antigen-presenting cells, which are crucial for the proper presentation of antigens involved in the immune response [69]. However, significant challenges remain. These include our incomplete understanding of ampullavirus immunology and its complex interplay with the immune system, the need for efficient and targeted delivery systems, and scalable and cost-effective production methods. This underlines the need for further research into the potential of ampullaviruses for vaccine development. If these challenges are addressed, it may be possible to develop effective vaccines targeting a wide variety of infectious diseases using ampullaviruses [70].

### 2.4 Other Biotechnological Applications

In addition to vaccine development, ampullaviruses can be used in biotechnological applications. Their special capsids are stable and well-engineered. They make them the top candidates to deliver vaccines for antigens. This delivery can produce an active immune response, as presented in Fig. (**2**). However, several challenges remain. Very little is known about these viruses, and it is not easy to culture them. However, the payoff of ampullaviruses for biotechnological applications remains a major issue. Their stable capsids and large genomes enable them to perform many functions. They pack a wide range of molecules to serve as vectors [71].

In contrast, ampullaviruses have huge benefits in biotechnology. However, there are several problems to solve before using them in biotechnology. It is hard to describe these viruses as they are new to science. This makes it hard to optimize their large-scale production. However, as they have stable capsids and large genomes, they are great for many biotech uses. Recent advances in research have improved methods and solved culturing problems. These advances may soon lead to wider use of ampullaviruses in biotechnological applications.

## 3 STRUCTURAL INSIGHTS

Ampullaviruses possess distinct features, such as a bottle-shaped head and tubular tail. Their unique structural characteristics raise intriguing questions in terms of assembly mechanisms and structural biology. Recent developments in imaging techniques, particularly cryo-electron microscopy, have significantly advanced our understanding of this virus family [72]. The study has aimed to elucidate the organization and function of the icosahedral head in encapsulating the viral genome and the tubular tail in host recognition and cell entry. These atypical structural features have heightened scientific curiosity regarding their assembly. Thus, unlocking the molecular details governing these atypical structures is of primary importance to researchers and biotechnologists.

Explorations of ampullaviruses in biotechnology have shown potential promise, tempered with challenges, which may likely be overcome as knowledge and technology advance, enabling their full utilization in diverse applications [73].

### 3.1 Advantages and Disadvantages

Although ampullaviruses hold huge potential for biotechnological applications, the benefits are yet to be optimized because challenges exist in implementing these viruses in biotechnological processes. One of the major challenges is that these viruses are hard to characterize, a factor that complicates their large-scale production optimization. Probably this is because they are relatively new in scientific research [27].

Despite these challenges, ampullaviruses have significant potential benefits for biotechnology due to their stable capsids and large genomes, rendering them very suitable for a wide range of biotechnological applications. Encouragingly, methodological advances in research and solutions to culturing challenges will soon enable broader utilization of ampullaviruses in biotechnological applications [74] (Table **1**).

#### 3.1.1 Advantages

1. Specific bacterial targeting: Ampullaviruses present an excellent feature; they can infect and lyse only specific bacterial hosts with very high specificity, which distinguishes them from other viral candidates. This high specificity makes them appealing for applications in the control of bacterial populations in both industrial and environmental settings [75].
2. Microbial ecosystem reshaping: The role of ampullaviruses in changing microbial ecosystems could be tremendous, as their targets are selective and specific to particular groups of host cells. This specificity can correct bacterial imbalances across a wide range of scenarios, thereby contributing to healthier microbial communities [76].
3. Prospects of nanotechnology: The “bottle-like” morphology of ampullaviruses indicates their potential applications in nanotechnology. These well-defined shapes can serve as templates for constructing new materials at the nanoscale, with customizable properties [37].

#### 3.1.2 Disadvantages

1. Narrow host range: The narrow host range of ampullaviruses is a major drawback. While their high specificity is advantageous for targeted applications, it can significantly limit their broader application in biotechnology. This specificity requires much consideration in the choice of target bacteria, and can be a limitation if the entire set of microbes needs to be addressed [77].
2. Safety concerns: Live viruses, even if host-specific, must be handled with stringent confinement measures and strictly observed safety protocols to avoid unintended consequences; this can also ensure the responsible use of ampullaviruses in research and industrial settings [78].

In conclusion, ampullaviruses possess fascinating structural features that are of great interest to virology researchers. Their unique bottle shape may hold a treasure trove of secrets regarding the assembly and function of the viral nanomachines. While these viruses show promising biotechnological applications, they should be approached with caution, considering their limitations, such as narrow host range and safety concerns. The ongoing exploration of ampullaviruses continues to reveal their relevance across a full spectrum of basic research studies within the broader biotechnological landscape.

The discovery of novel ampullaviruses that thrive under extreme conditions represents a significant advance in virology [78]. Organisms that have high adaptability under such challenging conditions not only enhance the knowledge of viral diversity, but also provide excellent prospects for biotechnological advancement. The characteristics of ampullaviruses from extreme environments to their roles in genomic sequencing and structural studies have potential applications in different areas. These include biofuel production, biopharmaceutical manufacturing, environmentally friendly biocontrol, and precision medicine.

## 4 AMPULLAVIRUSES: PIONEERS IN EXTREME ENVIRONMENTS

Ampullaviruses are relatively new to virospheres, but their ubiquity in extreme niches has drawn significant attention. Indeed, thriving at high temperatures, low pH, and other extreme environments, some ampullaviruses, such as the *Acidianus* bottle-shaped virus, exemplify fantastic adaptability and resilience [78]. Their unique morphologies, replication mechanisms, and interactions with hyperthermophilic archaea have provided valuable insights into viral survival and replication in extreme environments. The discovery of ampullaviruses has highlighted these elements, with the *Acidianus* bottle-shaped virus leading to an understanding of viral-host interactions in extreme environments [79].

A study by Kim *et al.* [80] described the role of family B DNA polymerase protein in replicating the ampullavirus genome. This protein replicates the spike forms, similar to other viruses related to caliciviruses, which infect halophilic archaea [80]. However, it is important to note that pleomorphic phages known as ampullaviruses were isolated from hot springs and showed activity against *Acidianus*, a member of the *Archaea* domain. This finding contrasts with the previous findings on halophilic archaea [81].

### 4.1 Genomic Sequencing: Unlocking the Genetic Toolkit

Genomic sequencing technologies have become essential to advancing ampullavirus research, providing researchers with an unparalleled level of detail in reading fine prints in ampullavirus genomes. The linear double-stranded DNA genome is approximately 23.8 kilobases long. It contains several open reading frames that form a bottle-shaped structure, which is a key feature of the genome, important for many biotechnological applications. The impact of sequencing extends beyond discoveries to significant profound biotechnological applications. This knowledge could improve targeted genetic engineering, potentially aiding the development of therapeutics for drug delivery, vaccines, and biofuel production.

Genomic sequencing technologies have proved to be an indispensable driver of advances in ampullavirus research, providing researchers with an unparalleled level of detail in ampullavirus genomes. The linear, double-stranded DNA genome is approximately 23.8 kilobases long [82]. This detailed genomic information has significant scientific and biotechnological implications. It could enhance targeted genetic engineering, thereby enabling the development of therapeutics for drug delivery, vaccines, and biofuel production. Continuous interdisciplinary research is essential to fully understand and exploit the biotechnological potential of ampullavirus genomes [83].

### 4.2 Structural Characterization: Inspiring Nanotechnology and Virology

High-resolution structural studies of ampullavirus capsids have revealed details responsible for their bottle-like shape and extraordinary structural stability. Such revelations have not only provoked curiosity in virology, but also may play a role in nanotechnology and material science [84]. Ampullaviruses, with their unique shapes, act as natural templates and have inspired innovations in drug delivery, sensor technology, and material engineering. Structural insights into ampullaviruses form a cornerstone for the development of antiviral strategies and the discovery of novel therapeutics. Research on ampullaviruses is now at the forefront of impacts on biotechnology, enhancing our genetic toolkit and pushing the frontiers of progress in nanotechnology and materials science [85].

### 4.3 Biofuel Production: Precision and Quality Enhancement

Tailored ampullaviruses are strategically engineered and precise in their design, targeting bacterial contaminants that affect biofuel production. These ampullaviruses have revolutionized biofuel production through selective detection and elimination of bacterial contaminants. Seamlessly integrated, these engineered viruses act as sentinels to optimize efficiency and increase biofuel yields, while ensuring high-quality products. Ampullaviruses have been engineered for use in this groundbreaking leap towards the precision, efficiency, and sustainability of biofuel production, and they show promise in contributing to the green energy sector [86,87].

### 4.4 Biopharmaceutical Production: Precision for Safety and Quality

Engineered ampullaviruses have shown promise in the manufacture of biopharmaceutical products due to their effective control over bacterial contaminants. Their precise targeting and neutralizing abilities have contributed to purity, consistency in bioproduction runs, efficiency gains, and safety [88]. The use of ampullaviruses represents a significant advance in controlling bacterial contaminants. This could help maintain product integrity and support sustainable, safe, and consistently high-quality output in this important sector [89].

### 4.5 Harnessing Ampullaviruses for Eco-friendly Biocontrol

Ampullaviruses have become important for controlling pests, pathogens, and deleterious organisms in diverse environments. Engineered ampullaviruses provide a sustainable and environmentally friendly alternative in cases where traditional chemical treatments are not practical or are harmful. The ability to precisely target deleterious bacterial populations in industrial processes or agriculture provides sustainability and environmentally responsible practices. This reduces reliance on chemical pesticides and introduces more precise biocontrol strategies [90,91].

### 4.6 Pioneering Precision Medicine: Ampullaviruses in Targeted Antibacterial Therapies

Ampullaviruses are potential candidates for directed antibacterial therapies. Their host specificity and ability to lyse specific bacterial hosts offer a new approach to the struggle against antibiotic-resistant bacteria [92]. Engineered ampullaviruses with high specificity could be used, minimizing harm to the patient's commensal bacteria. These viruses may also disrupt biofilms, potentially providing a new method for treating chronic and recurring infections [93]. Ampullaviruses are subjects of interest due to their extreme environmental applications, genomic complexity, structural characteristics, and potential uses in biotechnology and medicine. As research progresses, more innovative applications may emerge to utilize the unique characteristics of ampullaviruses. These applications could range from addressing environmental pollution to combating bacterial infections that have developed resistance to multiple drugs [92]. Ampullaviruses remain important in shaping the future of virology and the applied sciences to address diverse, complex challenges, as illustrated in Fig. (**3**).

## 5 EXTREME ENVIRONMENT ADAPTATION IN BIOTECHNOLOGY: UNLOCKING NATURE'S SOLUTIONS

Biotechnology remains an ever-changing field, continually inspired by nature's ingenious solutions. This review has highlighted the leading role of viruses, specifically ampullaviruses in extreme environments, in advancing the biotechnological revolution. Our comprehensive analysis has unveiled a new paradigm of Extreme Environment Adaptation (EEA), where nature's extremophiles and their viruses have been found to drive breakthrough biotechnological applications [94].

The peculiar type of virus, ampullaviruses, has been found to contribute massively to knowledge of the resilience mechanisms and represents a great reservoir of novel enzymes and biomolecules. These viral resources might revolutionize various industries, from biofuel production to nanotechnology. Thus, with increased elucidation of ampullaviruses, a surge in novel biotechnological applications is expected. Future research should focus on the following aspects:

1. Exploring the molecular basis of ampullavirus adaptations to extreme habitats.
2. Unraveling the full diversity of ampullaviruses through metagenomics and single-virus genomics.
3. Design of high-throughput screening protocols for the identification of novel enzymes and bioactive compounds from ampullaviruses.
4. Research on the ecological activities of ampullaviruses in extreme environments and their possible applications in bioremediation.

### 5.1 Principles of Extreme Environmental Adaptation in Biotechnology

Extremophiles are microorganisms that thrive in harsh environments, supporting EEA. These organisms possess unique biotechnological capabilities that are not yet fully explored due to their distinct biochemical pathways and molecular adaptations. Ampullaviruses, which have adapted to survive in extreme environments, are examples of such extremophiles [95]. These viruses contain highly specialized enzymes and structural proteins that make them resistant to extreme temperatures and pH levels, enabling their use in biotechnological applications. Extremozymes, highly effective enzymes resulting from adaptation to extreme environments, have become vital tools in various biotechnological processes. This demonstrates the enzymatic versatility of extremophiles and their potential for biotechnological advancement [96].

Among extremophiles, ampullaviruses excel at extreme environment adaptations of EEA, occupying a unique niche within the earth's most hostile abode. Their host specificity, biocontrol, and bioremediation potential, along with applications in precision medicine, underscore their importance in shaping the biotechnological landscape. The host specificity of ampullaviruses allows for sophisticated engineering applications. Engineered ampullaviruses can selectively target specific bacteria for industrial use, improving effectiveness while minimizing unintended effects [97]. Ampullaviruses offer environment-friendly biocontrol alternatives in various sectors, helping to manage harmful bacterial populations. They are used in waste treatment, agriculture, and bioremediation to address challenges sustainably. In medicine, the host specificity of ampullaviruses has potential applications in addressing drug-resistant bacteria. Engineered ampullaviruses could be designed to target specific bacterial strains, potentially reducing effects on a patient's overall microbiome [98]. In conclusion, ampullaviruses are notable organisms that adapt to extreme conditions, offering precise and sustainable solutions to all the challenges across various sectors.

### 5.2 Deciphering Host–Virus Dynamics: Unveiling Ampullaviruses in Extreme Environments

In virology, interactions between hosts and viruses significantly influence their biological and ecological effects. Understanding the mechanisms of ampullaviruses in extreme environments can reveal key mechanisms, which can further support fundamental research and advance biotechnological progress [99].

Host-virus interactions are central to virology, influencing viral biology and ecological impacts. These interactions help underlie the capacity of viruses to invade, replicate within, and successfully evade host immune responses [100]. The mechanisms governing host-virus interactions in extreme environments include host recognition, infection, replication, immune evasion, and lysis. Ampullaviruses have unique mechanisms that enable their persistence and survival in the hostile environments of biotopes [101]. Studying host-virus interplay in extremophilic environments has significant implications. A detailed examination of these host-virus interactions can lead to the development of modern biotechnology and offer valuable insights into the complex world of viral biology [101].

Extreme environments contain new genes, enzymes, and adaptations, which can lead to novel products and processes. Studies on host-virus interactions have uncovered new biotechnological features of natural extremophiles. Understanding host-virus interactions is crucial for tailoring viral properties. The precise design of viruses can be better used to execute different biotechnological processes that are more efficient and specific [102].

Precise viral design enables more efficient and specific biotechnological processes. Knowledge gained from host-virus interactions can provide applications in precision medicine, offering new therapies against antibiotic-resistant bacteria. Manipulating these interactions may reduce the effects on patient microbiota and help address some of the related modern medical challenges [103].

The precision with which ampullaviruses lyse the host bacteria has potential practical applications in biotechnological production. Better control over microbial contamination can improve product purity, reduce contamination control measures, and potentially shorten the time to marketing of the products. In-depth research into host-virus interactions can support scientific advances and directly influence biotechnology, forming the basis for innovative applications.

## 6 THE ENZYMATIC ARSENAL OF AMPULLAVIRUSES: A BIOTECHNOLOGICAL GOLDMINE

Resilient inhabitants of extreme environments, ampullaviruses represent a biotechnological goldmine due to their unique enzymatic arsenal. We have focused herein on the most promising applications of ampulla viruses in various industries, with particular attention to their enzymes as invaluable tools for high-temperature processes. Ampullaviruses have evolved genetic machinery to thrive under extreme circumstances and have developed enzymes adapted to high temperatures. These enzymes are becoming crucial tools in several biotechnological applications.

Key areas of applications include the following:

1. **Bioprocessing and Bioconversion:** They can be applied in the production of biofuels, bioplastics, and other bio-based products.
2. **Food and beverage industry**: They can be applied to the food and beverage industry, which can further ensure safety and quality optimization in high-temperature environments.
3. **Nanotechnology**: They possess potential applications in the synthesis and assembly of nanoscale materials.

The enzymes derived from ampullaviruses represent a convergence of nature's extreme adaptations and human innovation, leading to groundbreaking developments in biotechnology. As researchers work to unravel and harness the potential of ampullaviruses and their symbiotic relationship with extreme environments through biotechnology, they can pave the way for opportunities to address some of humanity’s most critical challenges, while simultaneously advancing scientific discovery.

Resilient inhabitants of the extreme environment, ampullaviruses have become a biotechnological goldmine due to their unique enzymatic arsenal. In this paper, we have focused on some of the most promising applications of ampulla viruses in various industries, with particular attention given to enzymes as invaluable tools for high-temperature processes [104]. Ampullaviruses are known to have evolved genetic machinery for thriving under extreme circumstances and develop enzymes adapted to high temperatures. Most of these enzymes are becoming crucial tools in several biotechnological applications [105]. Such enzymes hold promise for biotechnology because of the convergence of nature's most extreme adaptations with human innovation to groundbreaking development [106].

## 7 DECODING THE AMPULLAVIRAL ENIGMA: BRIDGING KNOWLEDGE GAPS

In gaining an understanding of ampullaviruses, several essential knowledge gaps remain in their biology, genomic diversity, ecological impacts, and biotechnological potential. While their resilience in extreme environments is well-documented, fundamental questions remain unanswered regarding the mechanisms of infection of ampulla viruses, strategies for viral replication, and their interaction with host organisms.

The extensive genomic diversity of ampullaviruses poses intriguing questions about the genetic elements driving their adaptability to extreme conditions [107]. Furthermore, the ability of ampullaviruses to influence microbial communities and nutrient cycling within extreme environments represents an exciting avenue for ecological research. This research can uncover the ecological effects of these viruses and their contributions to biogeochemical processes.

In addition, the biotechnological potential of ampullaviruses could open up novel opportunities for developing tools and high-temperature enzymes. A multi-omics approach involving genomics, transcriptomics, and proteomics, should be prioritized in studying these viruses to gain an in-depth insight into ampullavirus biology. Such research can unlock potential biotechnological and environmental applications [104].

## 8 INTERDISCIPLINARY EXPLORATION OF AMPULLAVIRUSES: UNLOCKING MYSTERIES AND INNOVATIONS

The discovery of ampullaviruses has led to interdisciplinary research, with extensive collaboration and knowledge transfer among various fields. At the interface between virology and extremophile biology, researchers are unveiling the ecological roles of ampullaviruses in extreme environments, revealing significant effects on the microbial community and biogeochemical cycling. Ongoing collaborations between virologists and biotechnologists are leading to further exploration of the biotechnological potential of ampulla viruses, from their identification to the application of high-temperature enzymes in various industries [27]. Researchers in virology, microbiology, and related fields are working together to understand the molecular adaptations of ampullaviruses explore the genetic elements behind their resilience, as well as study their interaction with host organisms. Additionally, interdisciplinary teams are investigating the ecological effects of ampullaviruses in extreme habitats, highlighting their ecological importance and contributions to biogeochemical cycles.

This comprehensive interdisciplinary study of ampullaviruses, within the framework of knowledge transfer and innovation, can bring together a variety of perspectives, leading to an enhancement of innovative approaches and the exploration of new research directions. These collaborations can ensure the translation of ampullavirus discoveries into practical biotechnological applications, benefiting industrial processes and providing environmental solutions. Moreover, interdisciplinary studies can also lead to a better understanding of extreme ecosystems and the underlying complex interplays among viruses, microbes, and biogeochemical processes [108]. In conclusion, ampullaviruses represent an interdisciplinary research subject, integrating diverse areas of science. Therefore, the unique adaptations of these organisms and their significant biotechnological potential could inspire a series of further investigations from researchers worldwide, leading to groundbreaking results and innovations.

## 9 CHALLENGES AND LIMITATIONS

The characterization of ampullaviruses is challenging due to the following reasons: these viruses very often have complex life cycles that involve multiple hosts or other environmental stages; hence, they are hard to culture and propagate in a controlled laboratory environment. The ampullaviruses' genomes can be highly variable; in fact, there exist species with segmented genomes or whose replication strategies are quite unusual. Moreover, many ampullaviruses are difficult to grow *in vitro*, which seriously hampers large-scale production and molecular studies at the desired level of detail [109].

These challenges in ampullavirus characterization thus have a huge bearing on the optimization of these viruses for large-scale production and biotechnological applications. Novel cultivation techniques to overcome such hurdles are based on the development of advanced genomic analytical tools [110].

While scattered in different ecosystems and presumably of great importance, ampullaviruses still stay enigmatic and elusive. In this regard, a number of factors complicate their investigation, which are described as follows: most of the ampullaviruses can persist under a very wide range of environmental conditions, really making it difficult to isolate and further research any single strain [111]. As interactions between ampullaviruses and their hosts are poorly understood, many complications occur with regard to the manipulation of these viruses for biotechnological purposes [101]. This high genetic diversity of ampullavirus populations also complicates the possibility of developing standardized research protocols and biotechnological applications. Such problems require interdisciplinary approaches, bringing together insights into virology, ecology, and bioinformatics, to delve deep into the complexities of ampullavirus biology.

In addition, their narrow host range, generally associated with safety considerations, limits the potential biotechnological application of ampullaviruses. A large number of ampullaviruses are very specific and narrow, which greatly limits their broad-spectrum applications [100]. Although rare, this ability to switch hosts raises concerns about the ecological impact ampullavirus application in biotechnology could have. In developing countries, work involving ampullaviruses for biotechnological purposes is positioned under strict biosafety regulations, which cannot be easily applied to large production scales [100].

These shortcomings have to be surmounted with caution; risk assessment and containment strategies can facilitate the safe use of ampullaviruses in biotechnological applications. Despite these challenges, ampullaviruses offer several opportunities for biotechnological applications. On one hand, some ampullaviruses are considered potential biocontrol agents against agricultural or invasive species [112]. Another interesting biotechnological application for ampullaviruses is the use of their unique structure for new gene delivery systems [27]. The possibilities of capsid self-assembly in ampullaviruses can also contribute to producing nanomaterials with a wide scope of applications [113].

Our research on ampullaviruses involves a minor limitation. There are no records or reports on the ethical use of the virus. It also lacks one of the societal implications of using such viruses for biotechnology; however, our study has focused on the biology and technology of ampullaviruses [114]. Nevertheless, we see the need to address ethical and social concerns. Future research may cover these concerns in detail, including the risks, benefits, and rules of use. Also, guidelines for ampullavirus use are crucial for safety and accountability. This can help to make informed decisions and build public trust in using these viruses in biotechnology.

This will, however, require further research on cultivation and characterization techniques and molecular mechanisms of host-virus interactions, engineering ampullaviruses with expanded hosts or improved safety profiles, and establishment of comprehensive frameworks for risk assessment of ampullavirus-based biotechnologies. Although ampullaviruses create serious problems in biotechnological applications, their specific features are also remarkable opportunities. Only when all challenges in the characterization of viruses, questions over their biology, and viral-safety considerations are fully overcome, is it possible to reach the true potential of these viruses for real applications in biotechnology. If new scientific knowledge continues to grow with technological know-how, then ampullaviruses could prove to be active tools in the fields from agriculture to biomedicine.

## CONCLUSION

This study has looked into the adaptability of ampullaviruses to extreme conditions, unveiling strategies of survival that could enhance the resilience of these microorganisms. This review has explored the critical host-virus interactions of ampullaviruses, shedding light on their ecological impacts and potential biotechnological exploitation. The rich genetic information on ampullaviruses could offer promising opportunities in biotechnology because of their high-temperature enzymes and special abilities. Notwithstanding this, the results have revealed gaps in knowledge that highlight the necessity of interdisciplinary collaboration among virologists, microbiologists, extremophile biologists, and biotechnologists. This collaboration could prove to be crucial for developing new approaches and breakthroughs, thereby increasing the chances of insightful and impactful discoveries. Overall, this review has provided all-inclusive coverage using terminologies suitable for researchers and experts in the field.

## Figures and Tables

**Fig. (2):**
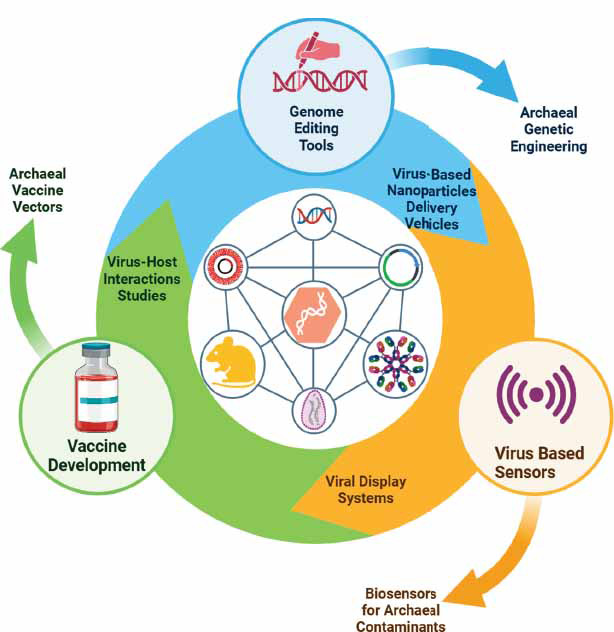
Schematic representation of some applications of ampullaviruses in archaeal virus research, including studies on virus-host interactions and viral display systems, providing new functions through genetic engineering. Other applications for these viruses include biosensing, vaccination, and genome editing.

**Fig. (3):**
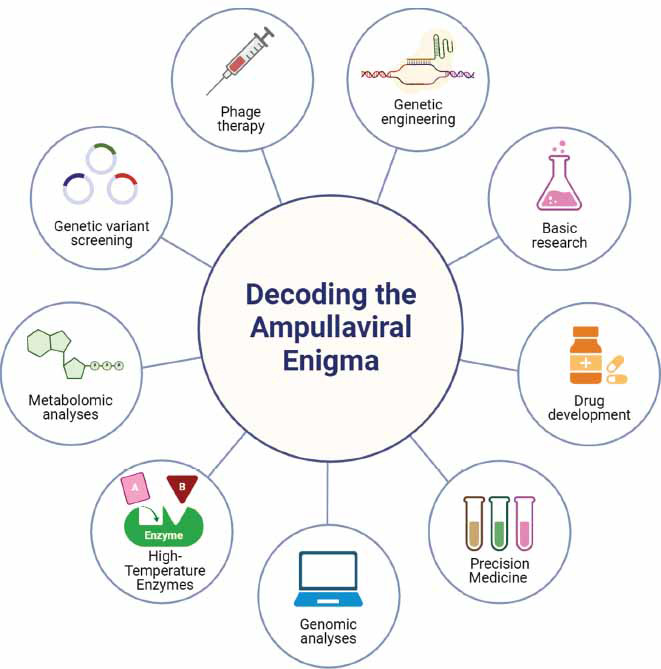
Schematic representation emphasizing the importance of decoding the ampullaviral enigma span from drug development to basic research, precision medicine, genomic analysis, high-temperature enzymes, metabolomic analyses, genetic variant screening, phage therapy, and genetic engineering.

**Table 1:** A detailed overview of the strengths, weaknesses, opportunities, and threats associated with the use of ampullaviruses for extreme environmental adaptation in biotechnology.

Strengths	Weaknesses
1. Extreme environment prowess: Ampullaviruses have evolved to thrive in extreme environments, such as acidic hot springs. Their ability to endure and replicate under such conditions makes them valuable candidates for biotechnology applications in similarly harsh settings.	1. Narrow host range: Ampullaviruses are highly specific to certain bacterial hosts. Their effectiveness is limited to these host species, which can be a drawback when dealing with diverse microbial populations.
2. Host specificity: Ampullaviruses exhibit high host specificity, targeting specific bacterial species. This specificity is advantageous in biotechnology, as it allows precise targeting of harmful bacteria while leaving beneficial ones untouched.	2. Limited genetic diversity: The relatively small number of known ampullavirus species may limit the genetic diversity available for biotechnological applications. This constraint could hinder the development of tailored solutions for specific biotechnological needs.
3. Potential for biocontrol: In extreme environments, where traditional chemical treatments may be impractical, ampullaviruses offer an eco-friendly alternative for controlling harmful bacteria. This can be particularly useful in industrial processes, like wastewater treatment.	3. New and unknown: These viruses are relatively new to science and there is still much unknown about them.
4. Structural stability: The unique bottle-like structure of ampullaviruses suggests structural stability, which could be leveraged for nanotechnological applications, such as constructing nanoscale materials with defined shapes.	4. Difficult to grow: Some viruses can be difficult to grow and produce in large quantities.
5. Unique ampulla-shaped capsids: These are stable and can be engineered to encapsulate a variety of different molecules with large genomes that can encode multiple genes.	5. Integration into host genome: Some viruses can integrate into the host genome, which can lead to long-term side effects.
**Opportunities**	**Threats**
1. Bioremediation: Ampullaviruses could be harnessed for bioremediation purposes in extreme environments polluted with harmful bacteria. This could have applications in environmental cleanup, especially in areas with geothermal activity.	1. Safety concerns: Working with live viruses, even host-specific ones, like ampullaviruses, poses inherent safety risks. Strict containment and safety protocols are essential to prevent accidental releases or unintended consequences.
2. Nanotechnology: The precise and unique shape of ampullaviruses could be exploited in nanotechnology for the design and assembly of nanoscale structures with specific properties.	2. Ethical considerations: The use of viruses, even for beneficial purposes, raises ethical questions about biocontrol and potential unintended ecological impacts.
3. Bioprospecting: Exploring extreme environments for new ampullavirus species may uncover novel genetic elements and adaptations that could be applied in biotechnology research.	3. Regulatory challenges: The regulatory landscape for using viruses in biotechnology applications can be complex. Navigating regulatory hurdles and ensuring compliance with safety regulations can be a significant challenge.
4. Investigation: Ampullaviruses are being investigated for use in new diagnostic and imaging tools, such as developing new ways to detect and diagnose diseases.	4. Public concern: Concerns exist about the safety of using viruses in biotechnology.

## LIST OF ABBREVIATIONS

DNA: Deoxyribonucleic Acid
VLPs: Virus-Like Particles
CPMV: Cowpea Mosaic Virus
ABV: Acidianus Bottle-shaped Virus
kb: Kilobase pairs
PCR: Polymerase Chain Reaction
CRISPR: Clustered Regularly Interspaced Short Palindromic Repeats
LAB: Lactic Acid Bacteria
CPB: Current Pharmaceutical Biotechnology
VNP: Virus Nanoparticles
dsDNA: Double-Stranded DNA
HPV: Human Papillomavirus
RNA: Ribonucleic Acid
ABV: Ampullaviridae
BMS: Biopharmaceutical Manufacturing System
